# Supplementary material for: Novel Oxygen- and Curcumin-Laden Ionic Liquid@Silica Nanocapsules for Enhanced Antimicrobial Photodynamic Therapy
Source: Pharmaceutics. 2023 Mar 28;15(4):1080. doi: 10.3390/pharmaceutics15041080 (PMC10140864; doi:10.3390/pharmaceutics15041080)
Supplement: Supplementary file 1 [file pharmaceutics-15-01080-s001.zip › pharmaceutics-2274360-supplementary.pdf]

SUPPLEMENTARY MATERIALS

Novel Oxygen- and Curcumin-Laden Ionic Liquid@Silica Nanocapsules for Enhanced Antimicrobial Photodynamic Therapy

Joana Henriques [1], João Pina [2], Mara E. M. Braga [1], Ana M. A. Dias [1], Patrícia Coimbra [1,*] and Hermínio C. de Sousa [1,*]

[1] Univ Coimbra, CIEPQPF, Department of Chemical Engineering, 3030-790 Coimbra, Portugal; joana.henriques@student.uc.pt (J.H.); marabraga@eq.uc.pt (M.E.M.B.); adias@eq.uc.pt (A.M.A.D.)

[2] Univ Coimbra, CQC-IMS, Department of Chemistry, 3004-535 Coimbra, Portugal; jpina@qui.uc.pt

* Correspondence: patricia@eq.uc.pt (P.C.); hsousa@eq.uc.pt (H.C.d.S.)

Table S1. Average hydrodynamic sizes of IL/water emulsions and of the correspondent nanocapsules (mean ± SD, n=3).

Sample	Z average size (nm)	PDI	Peak 1 (nm)	Peak 2 (nm)
CUR-[BMPYRR][NTf_2]/Water	283 ± 29	0.399 ± 0.023	343 ± 28	14 ± 1
CUR-[OMIM][NTf_2]/Water	207 ± 37	0.405 ± 0.001	285 ± 68	69 ± 16
CUR-[$P_{6,6,6,14}$][NTf_2]/Water	221 ± 3	0.278 ± 0.027	309 ± 11	-
CUR-[BMPYRR][NTf_2]@ncSi	2901 ± 479	0.947 ± 0.048	933 ± 83	167 ± 7
CUR-[OMIM][NTf_2]@ncSi	324 ± 42	0.425 ± 0.021	417 ± 76	89 ± 41
CUR-[$P_{6,6,6,14}$][NTf_2]@ncSi	394 ± 7	0.394 ± 0.009	532 ± 50	135 ± 22

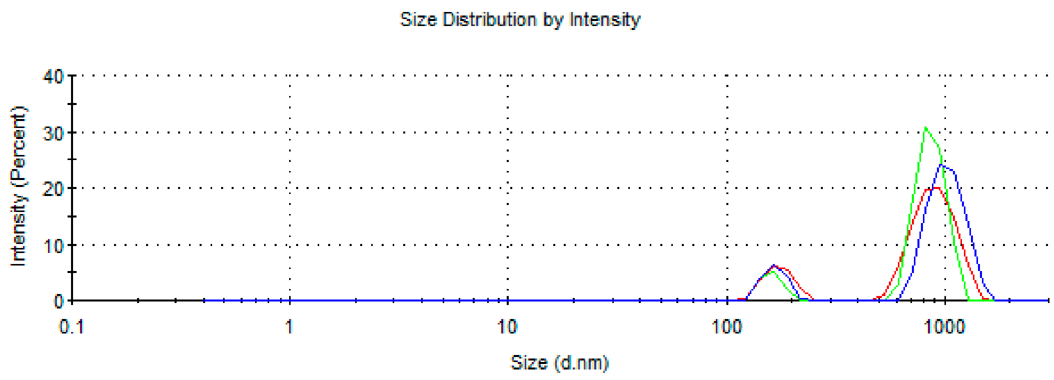

Figure S1. Size distribution of CUR-[BMPYRR][NTf_2]@ncSi nanoparticles

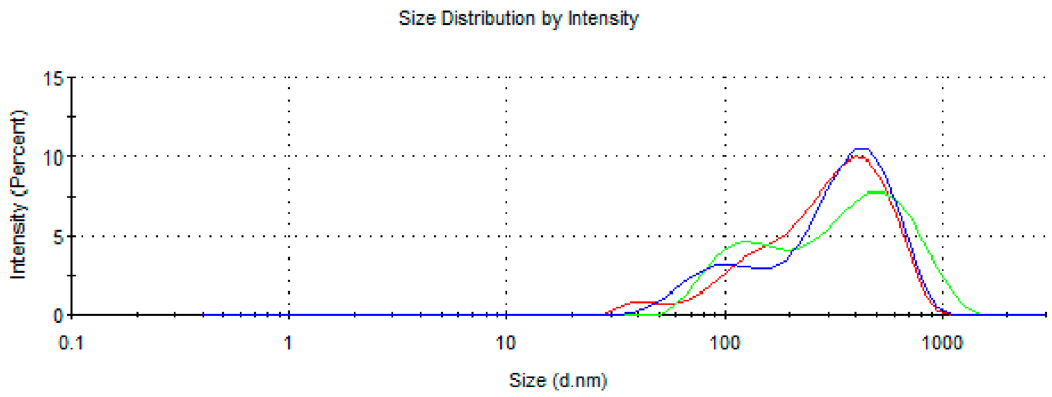

Figure S2. Size distribution of CUR-[OMIM][NTf_2]@ncSi nanoparticles

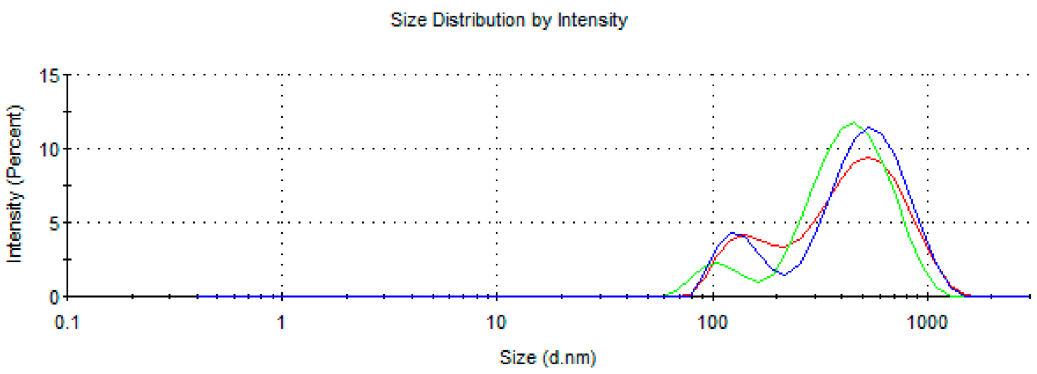

Figure S3. Size distribution of CUR-[$P_{6,6,6,14}$][NTf_2]@ncSi nanoparticles

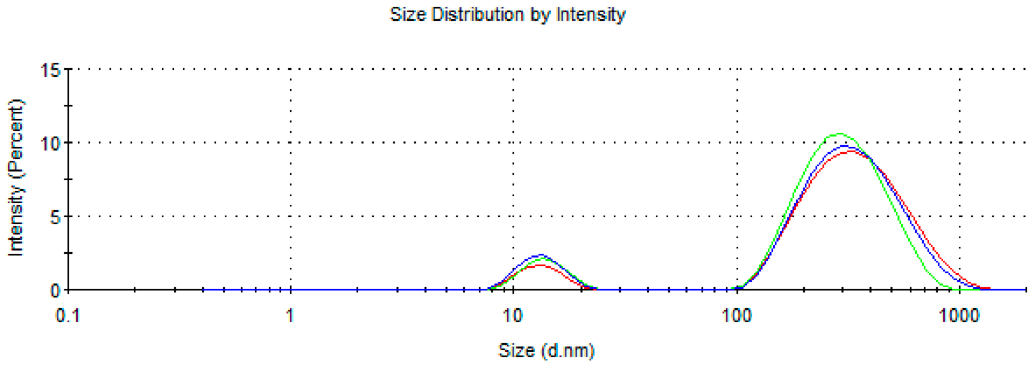

Figure S4. Size distribution of the IL droplets in the CUR-[BMPYRR][NTf2]/Water emulsion.

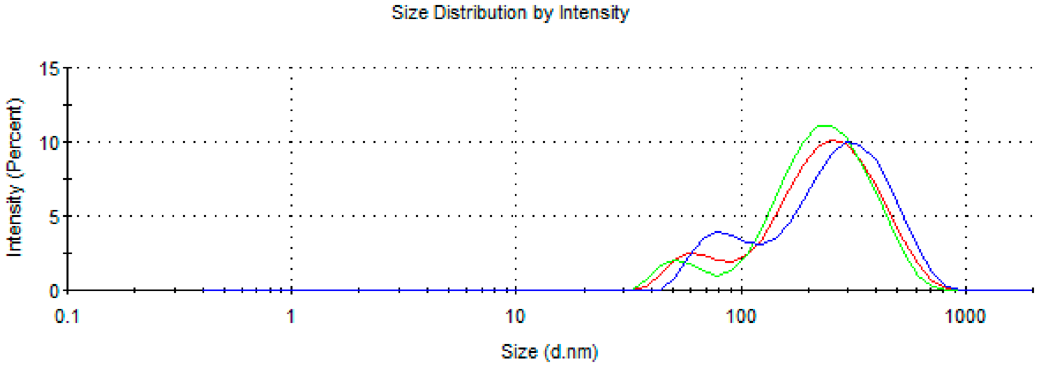

Figure S5. Size distribution of the IL droplets in the CUR-[OMIM][NTf2]/Water emulsion.

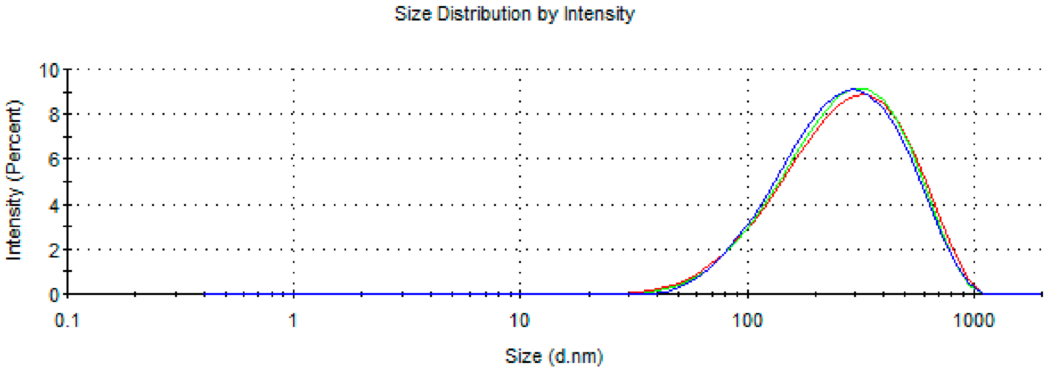

Figure S6. Size distribution of the IL droplets in the CUR-[$P_{6,6,6,14}$]][NTf2]/Water emulsion.

Table S2. Maximum concentration of dissolved oxygen (pO_{2Max}) and the correspondent time (t_{Max}) registered for the different samples. IL/water and nanocapsules/water overall volumetric mass transfer coefficients (K_La).

Sample	t_{Max} (min)	pO_{2Max} (ppm)	K_La* (h^{-1})
Water	2.5	5.4 ± 0.3	---
CUR-[BMPYRR][NTf_2]/Water	5.0	6.7 ± 0.5	58 ± 0
CUR-[BMPYRR][NTf_2]@ncSi	3.0	8.4 ± 0.5	127 ± 21
CUR-[OMIM][NTf_2]/Water	3.0	7.0 ± 0.2	157 ± 24
CUR-[OMIM][NTf2]@ncSi	2.0	7.7 ± 1.5	259 ± 3
CUR-[$P_{6,6,6,14}$][NTf_2]/Water	3.0	7.6 ± 0.8	167 ± 19
CUR-[$P_{6,6,6,14}$][NTf_2]@ncSi	2.5	8.1 ± 0.3	179 ± 39

*Calculated by plotting the initial linear segment of data represented in Figure 4 in the form of $\ln(pO_{2Max}/(pO_{2Max} - pO_2))$ vs time and by the performing of a linear adjustment. K_La corresponds to the slope of the fitted line.

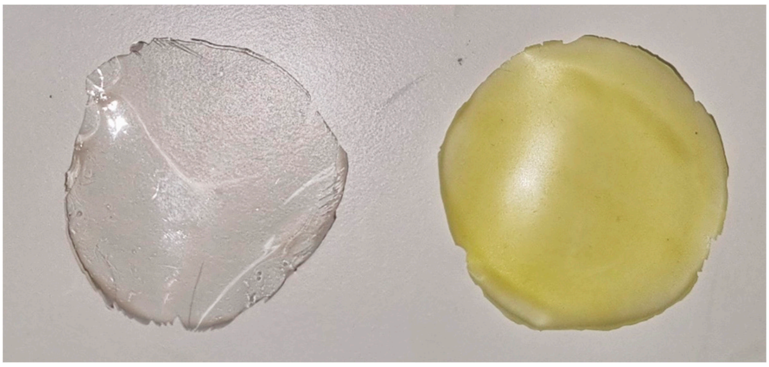

Figure S7. Gelatin film (left) and gelatin film loaded with CUR-[OMIM][NTf_2]@ncSi nanocapsules (right).

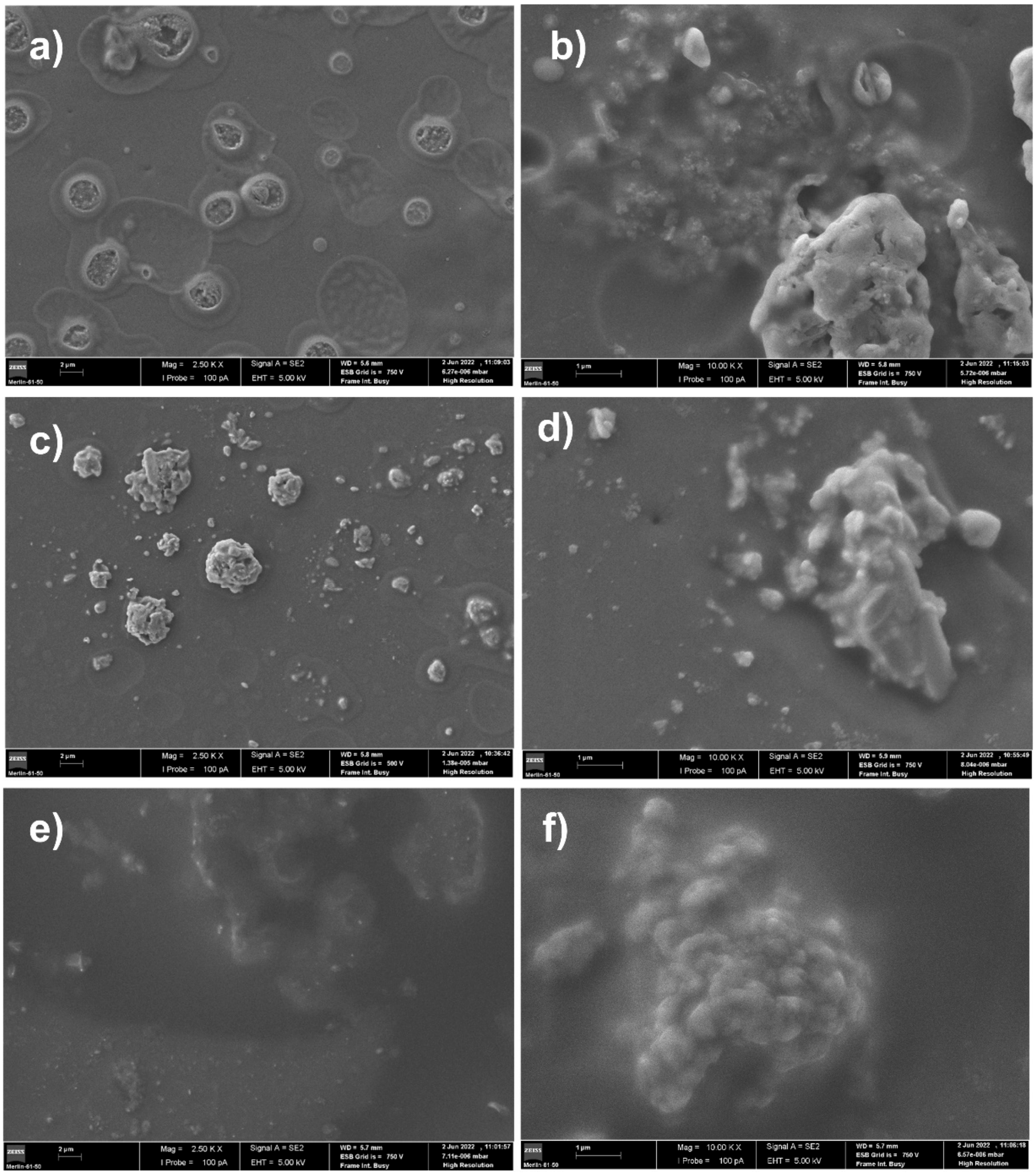

Figure S8. SEM images of the surface of gelatin films incorporating CUR-IL@ncSi nanocapsules: a), b) CUR-[BMPYRR][NTf_2]@ncSi; c), d) CUR-[OMIM][NTf_2]@ncSi; e) f) CUR-[$P_{6,6,6,14}$][NTf_2]@ncSi. Magnifications: 2500× (a, c, e); 10000× (b, d, f).
